# Simple Analysis of Gel Images With IOCBIO Gel Software

**DOI:** 10.21769/BioProtoc.5053

**Published:** 2024-08-20

**Authors:** Lucia Jaska, Rikke Birkedal, Martin Laasmaa, Marko Vendelin

**Affiliations:** Laboratory of Systems Biology, Department of Cybernetics, Tallinn University of Technology, Tallinn, Estonia

**Keywords:** Data analysis, Reproducibility, FAIR, Western blotting, Southern blotting, Isoelectric focusing, Python, Electrophoresis gel

## Abstract

Gel image analyses are often difficult to reproduce, as the most commonly used software, the ImageJ Gels plugin, does not automatically record any steps in the analysis process. This protocol provides detailed steps for image analysis using IOCBIO Gel software with western blot as an example; however, the protocol is applicable to all images obtained by electrophoresis, such as Southern blotting, northern blotting, and isoelectric focusing. IOCBIO Gel allows multiple sample analyses, linking the original image to all the operations performed on it, which can be stored in a central database or on a PC, ensuring ease of access and the possibility to perform corrections at each analysis stage. In addition, IOCBIO Gel is lightweight, with only minimal computer requirements.

Key features

• Free and open-source software for analyzing gel images.

• Reproducibility.

• Can be used with images obtained by electrophoresis, such as western blotting, Southern blotting, isoelectric focusing, and more.

## Background

When analyzing signal intensity from images, for example from western blots, researchers mainly use either proprietary software or the ImageJ Gels plugin [1]. Unfortunately, using those programs for image analysis contributes to a reproducibility crisis [2]. Proprietary software is closed source, so users do not know exactly how the analysis is performed, and the results can only be reproduced using the same software. While the ImageJ Gels plugin is open source, it does not automatically document the steps in the analysis process, leading to decreased reproducibility. IOCBIO Gel [3] solves both of these problems—it is free and open source, and it also records the original image together with all analysis steps performed on it, such as cropping, background subtraction, and lane selection. This makes it easy to reproduce the results and make corrections at any analysis stage. In addition, the software can store the data either on a PC or in a central database, facilitating efficient collaboration between researchers. Taken together, IOCBIO Gel is an efficient tool for image analysis, contributing to the implementation of FAIR (findability, accessibility, interoperability, and reusability) principles [4]. Further information can be found in the original article describing the software and testing its performance against the ImageJ Gels plugin [3].

## Equipment

1. Minimal requirements are mainly imposed by the corresponding operating system. The requirements of the application itself are minimal

## Software and datasets

1. IOCBIO Gel
  - Homepage: https://iocbio.gitlab.io/gel/
  - Downloads: https://gitlab.com/iocbio/gel/-/releases (version 1.0.3, release date July 11, 2024)
2. Video tutorials on YouTube, links also available on homepage
  - Installation
  - First start and setup
  - Basic tutorial
  - Advanced tutorial

## Procedure

- **Installation**
  1. Linux/Mac
    - To use the automatic installation script for Linux/Mac, first make sure that you have the latest pip installed by running:
      python3 -m pip install --user --upgrade pip
    - Then, open a terminal and go to the folder where you want to install the program. Then, run the following command:
      curl https://gitlab.com/iocbio/gel/-/raw/main/install.sh | bash
      or
      wget -qO - https://gitlab.com/iocbio/gel/-/raw/main/install.sh | bash
    - and run by:
      iocbio-gel/bin/iocbio-gel
  2. Windows
    Select the release under Releases and download Windows-executable packaged as a ZIP. Unpack the ZIP into any location of your PC and start the application by running gel.bat in the extracted folder. A video tutorial is available on YouTube and a written tutorial is available on homepage.
- **Running IOCBIO Gel**
  1. Starting the application
    - Linux/Mac users run the command in the folder that was used to install the software: iocbio-gel/bin/iocbio-gel
    - Windows users go to the extracted folder and run: gel.bat
    On the initial launch, you will need to choose the image source and the database, as described below under Settings.
  2. Settings
    When starting the application for the first time, you will be asked to choose a few settings. A video tutorial is available on YouTube. Settings are accessible in the main application window via a button on the left.
    First, select *Image source* and *Database connection*. Your selection depends on whether you want to store your files and results locally, i.e., on your own computer, or whether your files and results are stored in a shared space.
    - Image source
      Images can be stored and accessed either as files or through specialized image server interfaces.
      If you want to access your images as files, simply select *Local files* under *Image source selection*. This includes files stored on the local hard drives or files on some network-mounted filesystems, such as through network shares. It also allows you to use network storage and share the images in workgroups. The main requirement is that the images are visible through a local PC file path and are accessed in the same manner as local files.
      Your workplace may also have a specialized image database for central storage of microscopy and other images from all lab members. For example, in our laboratory, all images are stored in our OMERO central repository (https://www.openmicroscopy.org/omero/). In contrast to the local files, these central storage servers use specialized interfaces that can be used by the program to retrieve images.
      If your images and results are stored centrally in OMERO, select Image source *OMERO*.
      Insert:
      • The host name/address: should be available from your local systems administrator.
      • The port: you probably have to use the default port (4064).
      • Your username.
      • Your password.
    - Database connection
      Under *Database connection*, select *SQLite* if you want to store analysis data locally.
      Alternatively, if you have a central database, select *PostgreSQL* from the drop-down lists.
      Insert:
      • The hostname/address: should be available from your local systems administrator.
      • The port: we suggest using the default port (5432).
      • The SSL mode: we suggest using the default (prefer encryption).
      • The name of your database: should be available from your local systems administrator.
      • The schema: we suggest using the default (gel).
      • Your username.
      • Your password.
  3. View and edit modes
    The software has two modes to avoid accidental changes, viewing and editing (see the upper right corner, Figure 1). By default, the software opens in viewing mode. In order to make any changes, click on the toolbar button in the upper-right corner. It will change to editing mode, and you can add types of measurements, analyze gels, and perform other similar operations. Select the action you want to perform by pressing the corresponding button in the panel on the left, such as *Gels, Projects*, or *Types*.
    **Figure 1.**
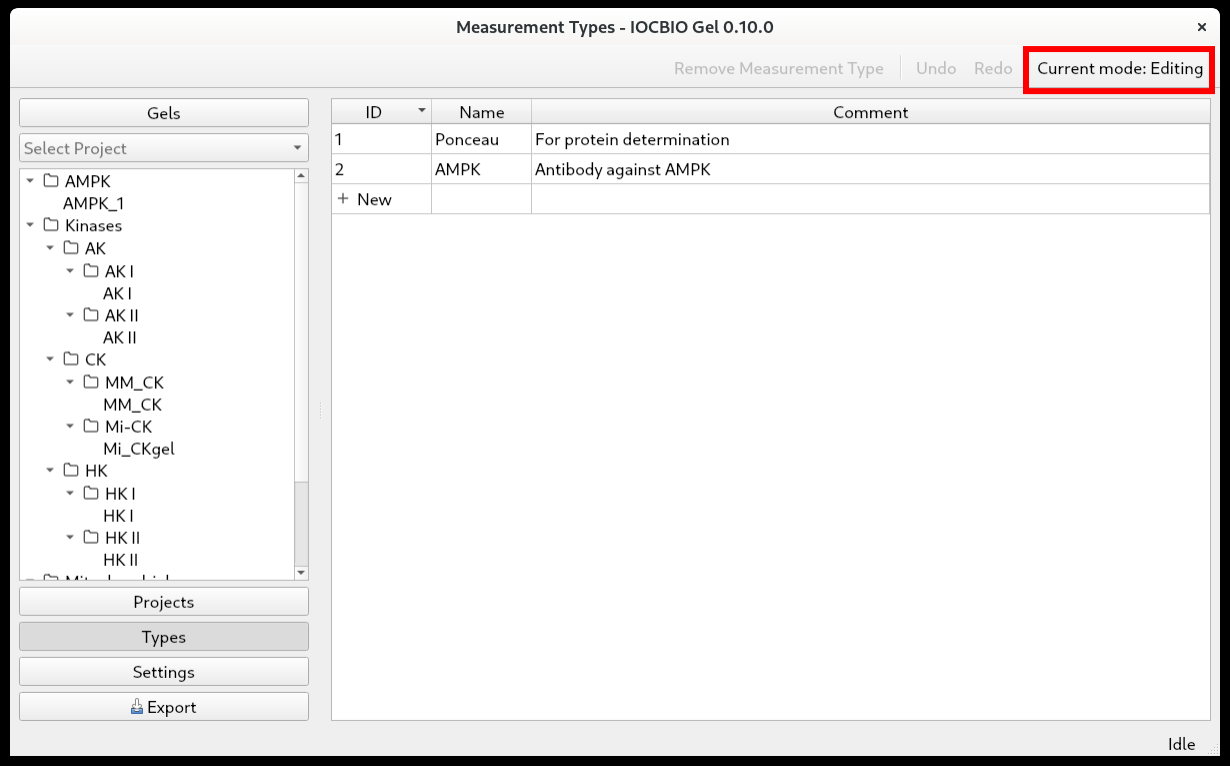
Edit and view mode shown while defining measurement types
  4. Types
    Under *Types*, you define the types of measurements. First, click on *Types* in the panel on the left. To add types, highlight the *Name* field and start typing. In the example shown above (Figure 1), we did a western blot to assess the expression of AMPK. Before antibody incubation, the membrane was stained with Ponceau to visualize and assess the overall protein content in each lane. Therefore, we put the types *Ponceau* and *AMPK*. With the types of measurements defined, continue with projects.
  5. Projects
    When doing multiple analyses, you might want to split your data according to which project they belong to. First, click on *Projects* in the panel on the left. Click *Add a new project*. By double-clicking on the *Name* and *Comment* fields, you can name the project and add a comment. Projects can be organized into a tree. For that, drag and drop the project under another one to form a branch. Notice that the subprojects are shown with the full path (the last column) consisting of the parent and the child names (Figure 2).
    **Figure 2.**
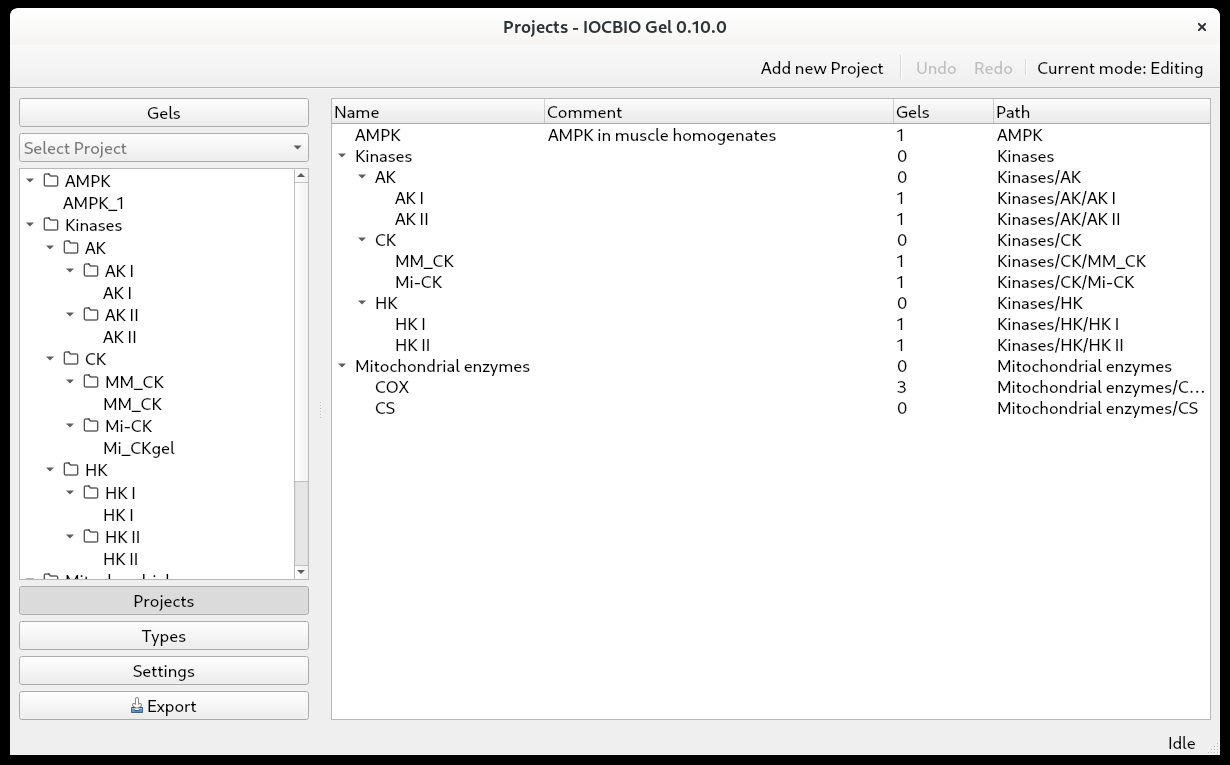
Projects can be defined as a hierarchy
  6. Gels
    First, click on *Gels* in the panel on the left. Define a new gel by clicking on *+ New* in the table. That is possible only when the application is in the editing mode.
    Name the gel: Highlight the *Name* field and start typing.
    Set the reference time. In our case, we used the date of transfer as the reference time, but this is open to interpretation, and each user can decide which reference time to use. Highlight the *Date and time* field and double-click to see the drop-down calendar, where you can select the correct date. You can also specify the time of the day. If this field is left blank, then the software will automatically put the current time.
    Insert a comment, if needed, by highlighting the *Comment* field and typing.
    Here, you can also select which project this gel belongs to. Double-click on the *Projects* field and select the project.
    The number of lanes can be set later and is shown based on the current records available for this gel.
    You are now ready to start analyzing your images.
  7. Image analysis
    There is a basic and advanced usage video tutorial available on YouTube.
    Clicking on *Gels* shows you a list of all the gels you have. To start the image analysis of a particular gel, press its ID in the list of gels or the name of the gel in the left column.
    Define the lanes of the gel. Press *+ New* until you have the number of lanes that you want to analyze.
    In our lab, we often have two ladders—one in the left-most and one in the right-most lane. As we do not include the ladder in the image analysis, we only analyze, for example, 13 out of 15 lanes (Figure 3). It is up to you how to define lane indexing, whether to take into account ladders or not.
    There are multiple columns:
    • ID: the ID of the lane
    • Lane: the number of the lane
    • Sample ID
    • Protein: the amount of sample protein that was loaded into each well on the gel
    • Reference: tick if the samples are used as references for comparing between gels (could be one or multiple)
    • Comments
    Notice that each lane has two IDs. Lane ID, marked as ID in the software, is a unique ID for each lane among all gels in the local database. This is generated by the software and does not have to be changed by the user. Sample ID allows you to link the sample in this lane to your other records. It can be either alphanumeric or numeric, and advanced users can impose more restrictions on it by changing the database rules directly using database software. Through the sample ID, you can also link your gel measurements to the data from other experiments in the database if you have other software using the same database. In our case, we keep animal lineage, sample descriptions, and number of experimental records all stored in the same database and analyzed or entered through an open-source web interface or specialized software for analysis of time series or calcium sparks [5,6].
    If you want to shift the number of lanes, it is easier to do this already after adding the first lane. The lane number will increase with each new addition.
    Note that you can sort the lanes in ascending or descending order. By sorting in descending order, you can keep adding new lanes on the top of the table.
    When the details of the different lanes are added, you can add the images from this gel. Click on *Add new image*. If you did multiple images of the same membrane, for example, Ponceau and antibody staining, both images are added here. You are now ready to analyze the signal intensity of the lanes in the pictures.
    **Figure 3.**
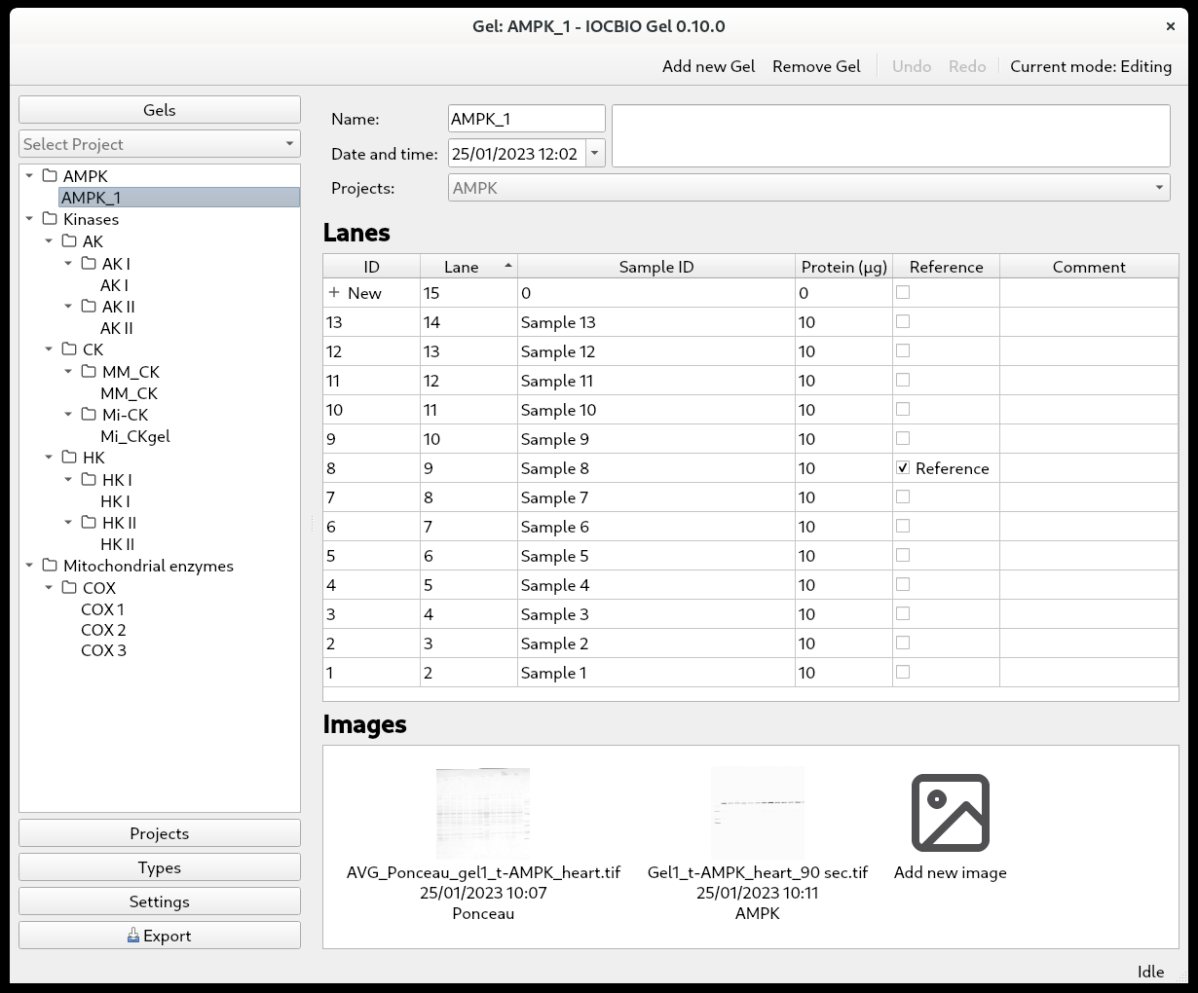
Overview of a single gel with its lanes and connected images
    - Image selection
      With the gel of interest selected, double-click on the image you want to analyze under *Images.* This will open the picture in the *Adjust* tab. Before that, there is the *Raw* tab, where you see the raw image as well as the image without the applied colormap.
    - Defining the region of interest (ROI)
      In the *Adjust* tab, click on *Add ROI* to select the ROI for your analysis. The ROI will be marked with a red square. The ROI can be moved by clicking within the ROI and dragging it to where you want it to be.
      The ROI has two handles. In the upper-left corner, there is a circular handle for changing the rotation of the ROI. In the lower-right corner, there is a diamond-shaped handle for changing the size of the ROI. The rotation can also be set on the slider in the upper right corner. When you have defined your ROI, click *Apply*.
    - Background subtraction
      After defining the ROI, you will automatically be taken to the *Background* tab, where you can subtract the background. First, you must define whether the background color is dark or light. Then, you select the kind of background subtraction that suits your image. Three kinds of background subtractions are available: *Flat, Ball*, and *Ellipsoid*.
      Select the kind of background subtraction most suitable for your image. If the background has little variation, select the flat background. If the background is lighter in one part of the image than another, select *Ball* or *Ellipsoid*. For the latter two options, you need to define the radius of the ball or the radii of the ellipse. Select a relatively large radius (for example, 500 or more). If the radius is too small, part of the signal will also be subtracted. Click *Apply* to see the background that was subtracted as well as the final result. If you are unhappy with the result, try out another kind of background subtraction or change the diameter used for the background subtraction.
    - Marking and positioning the lanes for analysis
      With the background subtracted, go to *Lanes* and click *Add new lane*. Position the lane on your image. Click *add new lane* and position the second lane as well. After that, every new lane will be positioned automatically based on the location of the previous lanes. Add new lanes until you have marked all the lanes that you want to analyze with an analysis lane. In each analysis lane, the center is marked with a green dotted line, and the sides are marked with green dashed lines. The intensity profile of each analysis lane will show up in the right-side panel (Figure 4).
      You can zoom in and out by clicking on the gel picture and using the scrolling wheel of your mouse.
      You will need to adjust the position and shape of the analysis lanes.
      To move an analysis lane, position the mouse over the central dotted line—it will turn to a solid, red line. Click to drag and drop.
      To change the width of the analysis lane, position the mouse over the little red square on the right side of the lane; this little square will turn yellow. Click and drag to adjust the width of the lane.
      You can choose to adjust the width of just one analysis lane at a time or to keep the same width of all analysis lanes. For this, there is a toggle button in the upper-right corner, where you can choose *Lane widths: Individual* to change the width of one analysis lane, or *Lane widths: Synced* to keep the same width of all analysis lanes.
      If the lane on your image is curved, you can incline or curve the analysis lane to follow the lane on the image.
      To incline the analysis lane, the central line has a top and a bottom handle. They are marked as little red squares, and they can be moved left or right to incline the analysis lane according to the lane in your picture.
      To curve the analysis lane, double-click on the central line to add extra handles. They will appear as little red squares along the central line, where you double-click. To grab a handle, position the mouse over it, and it will turn yellow. Click to grab and drag the handles on the analysis lane to follow the shape of the lane on the image. You can add multiple handles, but you must move the handle you made before you can add an additional handle (Figure 4).
      All actions can be reversed by pressing *Undo* in the toolbar (shortcut Ctrl + z).
      To remove a handle, place the mouse over the handle and check that the little red square changes color to yellow. Then, right-click on the handle and choose *Remove handle* from the drop-down menu. The analysis lane will straighten between the remaining handles.
      When all the analysis lanes are adjusted according to the picture, you can examine the intensity profile for each lane.
      **Figure 4.**
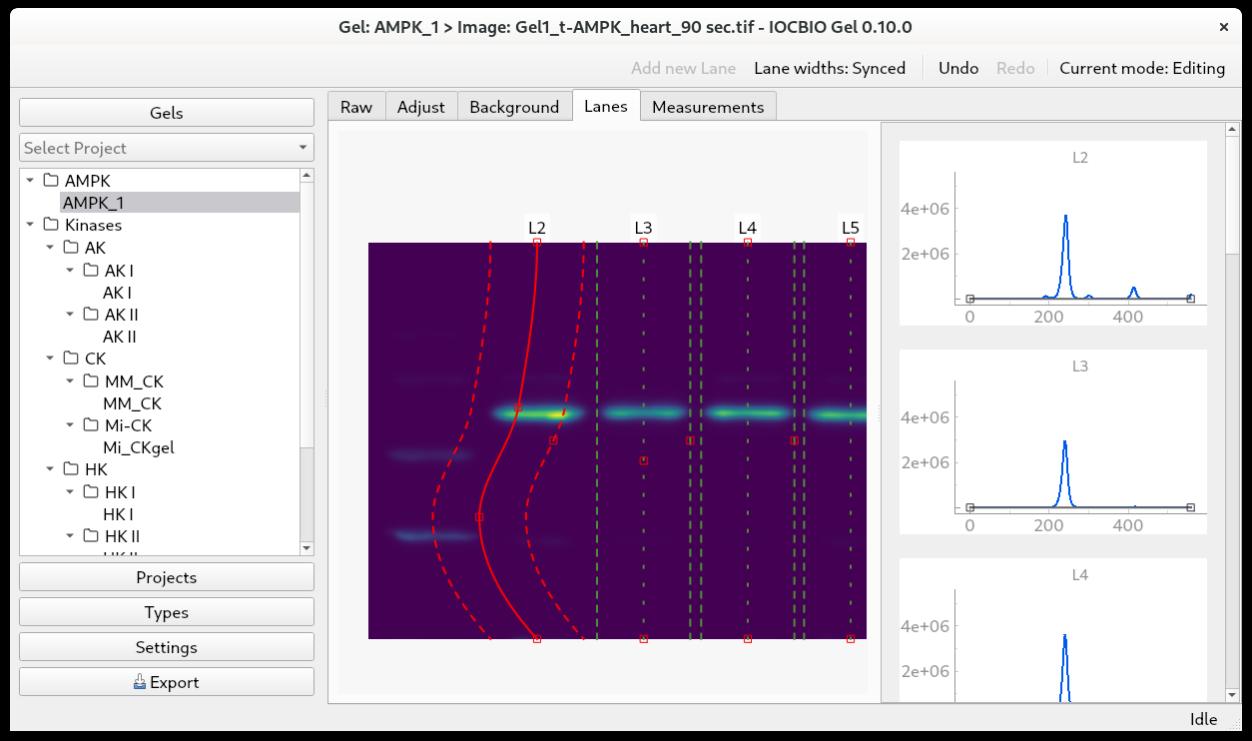
Gel lanes on gel image
    - Setting the baseline of the intensity profile
      The intensity profile of each lane is shown in the right-side panel (Figure 4). Here, it is possible to adjust the position of the baseline. We usually adjust the baseline to follow the dips of the intensity profile.
      To interact with a plot, you have to activate it. To do that, click on it. The plot will be deactivated automatically as soon as the mouse pointer moves out of the plot area.
      You can zoom in and out by clicking on the intensity plot and using the scrolling wheel of your mouse. To return to the original view, you can press the A button in the lower-left corner of the plot.
      To add a handle, double-click on the baseline, and the handle will appear as a little black square.
      To grab a handle, click on the window to ensure it is active. Place the mouse over the handle and check that the little black square becomes bold. Then, you can click and drag to position it.
      To remove a handle, place the mouse over the handle and check that the little black square changes to bold. Then you can right-click on the handle and choose *Remove handle* from the drop-down menu.
      When you are happy with the position of the baseline in all the intensity profiles, you can move on to the *Measurements* tab.
    - Obtaining signal intensity measurements
      On the *Measurements* tab, you first add a new measurement. The measurement corresponds to the integrated signal intensity over the region of interest. Within the family of methods analyzed by IOCBIO Gel, it is assumed that the measurement of interest *m* is given by the formula:
$$m=\int_{a}^{b}\left(I\left(x\right)-I_{bg}\left(x\right)\right)dx$$ (1)
      where *x* is a position along the intensity profile of a lane of interest, *I(x)* corresponds to intensity at position *x*, and *I_bg_(x)* is the baseline intensity. Note that since the pixel intensities are unitless and determined by specific camera hardware and other conditions, the measurement result *m* is also unitless. Therefore, *m* should be used in subsequent analysis only for relative comparisons with other measurement results, typically by dividing the compared measurements by a reference value.
      To add a measurement, you can either click *Add new measurement* in the upper-right corner or click + *New* in the lower-right corner (Figure 5). Next, select the type of measurement that you are doing by double-clicking on the *Type* field and selecting it from the drop-down list. In the example shown below, we selected Ponceau.
      **Figure 5.**
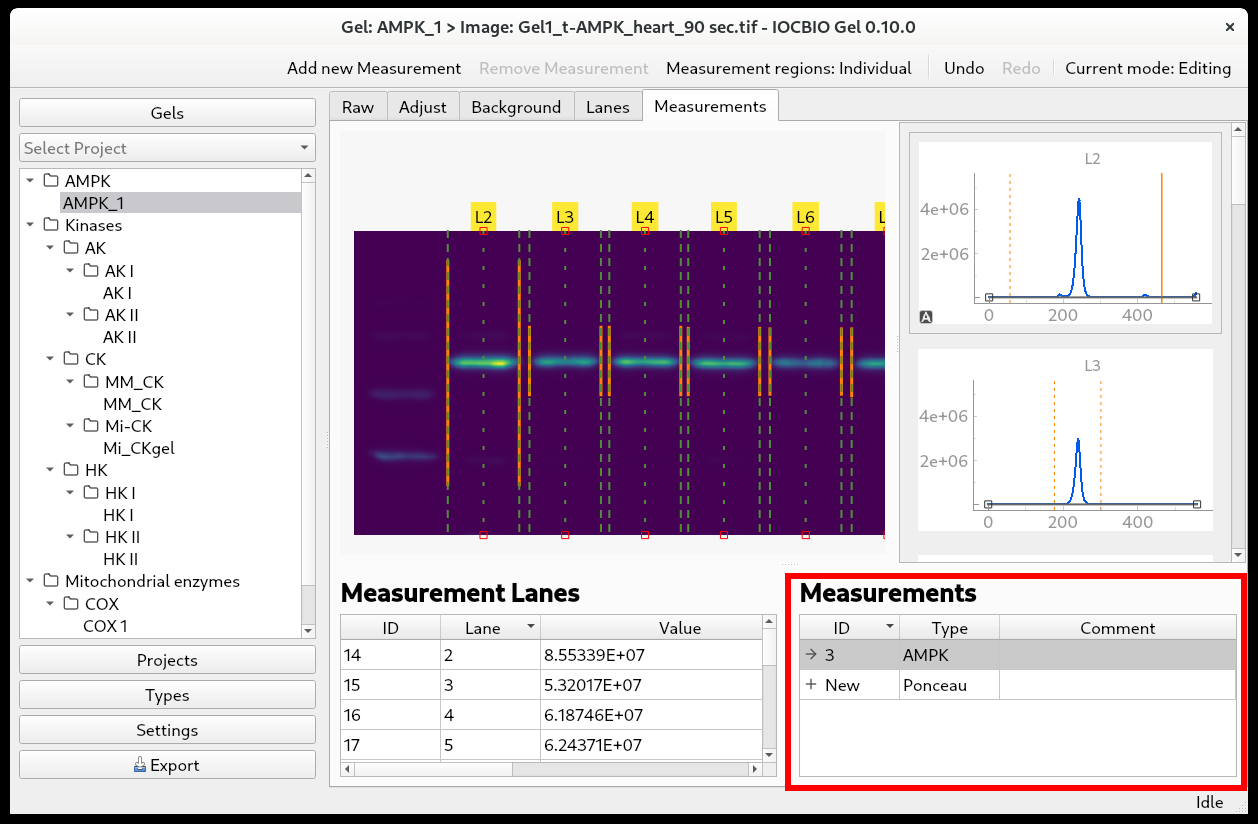
Selecting measurement type for an image (bottom right) and adjusting the region where the integration is done (top-right graph)
      Now, you simply click with the mouse on all the lanes that you want to use for your analysis. The dashed green lines marking the edges of each lane will be highlighted by a solid orange line below, and the intensity value will be shown in the *Measurement Lanes* field below the image. In this field, each measurement is shown with:
      • Its own ID.
      • The lane number.
      • The value of the intensity.
      • Success—tick if the measurement is successful.
      • Comments—if you have any.
      It is quite common that you do not want to measure the intensity of all the bands in each lane, but rather one or—in the case of protein staining—some of the bands, i.e., you want to specify the height of your analysis lanes. To select the band(s) whose intensity you want to measure, go to the intensity profile for each lane. There are sliders at each end of the profile, which are displayed as yellow dashed lines. Click on the intensity profile to activate the window and position the mouse over the slider; it will turn into a solid, yellow line. Click with the left mouse button and hold it down while adjusting the slider’s position. Notice that the value in Measurement Lanes changes as you adjust the sliders.
      You can choose to adjust the height of just one analysis lane at a time or to keep the same height of all analysis lanes. For this, there is a toggle button in the upper right corner, where you can choose *Measurement regions: Individual* to change the height of one analysis lane, or *Measurement regions: Synced* to keep the same height of all analysis lanes.
      In the example shown, we uploaded two images from the same membrane: In one image, the overall protein was stained using Ponceau, and in the other image, the protein of interest was labeled with antibodies.
      After analyzing the overall protein stain intensity in each lane, go back to *Gels* (in the upper-left corner) and click on the ID of the gel you are analyzing.
      Double-click on the next image that you want to analyze. In this example, it is the image with the protein of interest, t-AMPK.
      Adjust the ROI of the image (see step B7b) and analyze this image as before, subtracting the background, marking the lanes for analysis, setting the baseline of the intensity profile, and obtaining signal intensity measurements.

## Data analysis

Statistical analysis should be performed in another application. You can choose to directly fetch the data from the database or export it into a spreadsheet first.

1. Accessing the data from the database directly
  Many of the statistical analysis programs or environments support connection to SQL databases. Those include R and Python. Depending on the environment, you have to define the connection to the database and fetch the data using an SQL script. Two example SQL scripts are available at main/sql. Example code for data fetching in R is available at iocbioR.
2. Exporting the results
  As an alternative to fetching the data from the database directly, you can export it to a spreadsheet. To do so, click on *Export* in the lower-left corner. You will be prompted to name the file and select a location for your exported data. The Excel file has several sheets with the information from the gel analysis software:
  • Gels
  • Measurement types
  • Gel lanes
  • Images
  • Measurement (raw)
  • Reference measurement
  • Measurements (normalized)
  For most users, the last sheet with the normalized data will be the most interesting. It shows the measurement intensity values normalized to the reference sample that was defined when specifying the number of lanes to analyze (see *Image analysis*). If you have more than one reference lane, the values from each lane will be normalized to an average of the values from the reference lanes. These normalized values take into account the protein content that was specified for each of the lanes.
  The normalization is only within one type and within one gel. Thus, the overall protein stain intensity (in this example by Ponceau, P) is normalized to the protein stain intensity of the reference sample (Pref), i.e., P/Pref. The signal intensity of the protein of interest (S) is normalized to the signal intensity of the reference sample (Sref), i.e., S/Sref.
  To analyze the protein of interest, it is common practice to relate its intensity to the overall protein stain intensity of each lane. Thus, you compare S/P between lanes within one image.
  However, it is common to have so many samples that you want to compare between images as well. To compare between images, you must calculate S/Sref/P/Pref.
  If you have only a few images to compare, this can easily be done in Excel. However, for larger datasets, we recommend using a database so these ratios can be calculated in the SQL command to fetch the data.

## Validation of protocol

This protocol or parts of it have been used and validated in the following research article:

- Kütt et al. [3]. Simple analysis of gel images with IOCBIO Gel. *BMC Biology* (Figure 3, panels B–C).

## General notes and troubleshooting

**General notes**

1. Limitations of the software.
  Currently, setting the baseline of the intensity profile has to be done individually for each lane. We plan to add a syncing feature, as in Lane width or Measurement region selection. We are also working on improving the export feature to include the export of the database snapshot to share with other research teams.
2. Bugs and feature requests.
  IOCBIO Gel is an open-source software and relies on feedback from users in its development. This includes new features and fixing bugs.
  Bugs and new feature requests are considered as “Issues” and are reported on the project page. To see current issues and report new ones, follow the link *Issues* on the project page. Project is hosted at GitLab. If you want to open a new issue or comment on the reported one, you have to register as a user on that platform.
  To open a new issue, press the button *New issue* on the GitLab issues page.
  Next:
  • Write the title of the issue.
  • Keep Type as the default, “Issue”.
  • Write a description of the issue.
  • Assignee, milestone, and labels do not have to be selected.
  • Press the blue button *Create issue*.
  While filing the issue, feel free to paste screenshots that describe the problem. These include screenshots with the error messages and configuration settings. While care has been taken to avoid showing database login passwords in the configuration settings, please check all the screenshots for sensitive information before pasting them into GitLab.

**Troubleshooting**

Problem 1: Error while installing ZeroC ICE

Possible cause: OMERO Python library requires a specific version of ZeroC ICE. At the moment of writing, 3.6.5.

Solution: That version is not possible to install with Python 3.11 and newer due to a bug. Fixes have been available but for the newer ZeroC Ice version (https://github.com/zeroc-ice/ice/pull/1394). We have backported these bug fixes to 3.6.5 and made the patched version available in a separate repository. It can be installed using the following URL: https://gitlab.com/iocbio/libs/zeroc-ice-py/-/archive/v3.6.5/zeroc-ice-py-v3.6.5.tar.gz.

Problem 2: Background subtraction not working properly.

Possible cause: The original image-capturing software changed the background, for example from dark to light.

Solution: Try with a dark background if the subtraction does not work with a light background or the other way around.
